# Case Report: Jet lag and travel fatigue effects on performance in a world-class paralympic shot-putter after eastward long-haul transmeridian travel

**DOI:** 10.3389/fspor.2026.1745296

**Published:** 2026-02-10

**Authors:** Exal Garcia-Carrillo, Rohit K. Thapa, Felipe Hermosilla-Palma, Miguel Alarcón-Rivera, Claudio Farías-Valenzuela, Jairo Azócar-Gallardo, Rodrigo Yáñez-Sepúlveda, Jorge Olivares-Arancibia, Antonio Castillo-Paredes, Julio Calleja-González, Lawrence W. Judge, Héctor Fuentes-Barría, Júlio B. Mello

**Affiliations:** 1 Department of Physical Activity Sciences, Faculty of Education Sciences, Universidad Católica del Maule, Talca, Chile; 2 Department of Physical Activity Sciences, Universidad de Los Lagos, Osorno, Chile; 3 Symbiosis School of Sports Sciences, Symbiosis International (Deemed University), Pune, India; 4 Pedagogía en Educación Física, Facultad de Educación, Universidad Autónoma de Chile, Talca, Chile; 5 Escuela de Ciencias del Deporte y Actividad Física, Facultad de Salud, Universidad Santo Tomás, Talca, Chile; 6 Escuela de Ciencias de la Actividad Física, el Deporte y la Salud, Universidad de Santiago de Chile (USACH), Santiago, Chile; 7 Programa de Investigación en Deporte, Sociedad y Buen Vivir (DSBv), Universidad de Los Lagos, Osorno, Chile; 8 Faculty of Education and Social Sciences, Universidad Andres Bello, Viña del Mar, Chile; 9 School of Medicine, Universidad Espíritu Santo, Samborondón, Ecuador; 10 Grupo AFySE, Investigación en Actividad Física y Salud Escolar, Escuela de Pedagogía en Educación Física, Facultad de Educación, Universidad de Las Américas, Santiago, Chile; 11 Department of Physical Education and Sport, Faculty of Education and Sport, University of the Basque Country (EHU), Vitoria, Spain; 12 School College of Health and Human Services, Florida Gulf Coast University, Fort Myers, FL, United States; 13 School of Kinesiology, Ball State University, Muncie, IN, United States; 14 Vicerrectoría de Investigación e Innovación, Universidad Arturo Prat, Iquique, Chile; 15 Escuela de Odontología, Facultad de Odontología, Universidad Andres Bello, Concepción, Chile; 16 School of Physical Education, Pontificia Universidad Católica de Valparaíso, Valparaíso, Chile

**Keywords:** adaptive sports, athletic performance, jet lag symptoms, para-athletes, physical fitness, sports, sports for persons with disabilities, track and field

## Abstract

**Introduction:**

This case report aimed to examine the effects of long-haul eastward transmeridian travel on neuromuscular performance, subjective well-being, and internal training load in a World-class female Paralympic shot-putter preparing for the Paris 2024 Paralympic Games.

**Methods:**

An F54-class athlete was monitored for 21 days surrounding a flight from Chile to France (eastward, six time zones). Daily assessments included handgrip strength (HGS), shot-put throwing distance, subjective well-being, and session rating of perceived exertion (s-RPE). Descriptive statistics and Spearman's correlation were used to explore relationships between performance and physiological/subjective variables.

**Results:**

Transient reductions in HGS occurred for 3–4 days post-travel, followed by daily fluctuations. Jet lag and fatigue scores were elevated during the first post-travel days but showed progressive improvement, returning to near-baseline levels by approximately two weeks before competition. Throwing performance showed a moderate positive correlation with left-hand HGS (*ρ* = 0.52, *p* = 0.032) and a negative correlation with s-RPE (*ρ* = –0.56, *p* = 0.018). Gastrointestinal disruptions, particularly in stool frequency and consistency, persisted longer than other subjective variables.

**Conclusion:**

Long-haul transmeridian travel was associated with transient neuromuscular and perceptual disturbances in a world-class Paralympic athlete. HGS and s-RPE emerged as practical and responsive markers of travel-induced fatigue and performance readiness during the 21-day period surrounding international travel. These findings highlight the need for individualized monitoring strategies during the travel adaptation period in elite Paralympic sport contexts.

## 1 Introduction

Elite athletes frequently travel internationally, often across continents, to participate in major tournaments (1). These travels for international competitions usually requires crossing several time zones referred as transmeridian travel (distinguished as eastward or westward based on direction) (2). This is often, but not exclusively, associated with long-haul flights, a broader term for extended journeys (3). Such long travel frequently induces two primary, though different, conditions: travel fatigue and jet lag (3). Travel fatigue is a broader, non-circadian phenomenon arising from the general stressors of travel (e.g., cramped seating, sleep disruption) and can occur even without time-zone crossing (4). In contrast, jet lag is explicitly a circadian rhythm disorder caused by a desynchronization between internal biological rhythms and the external environment (i.e., circadian rhythm disorder) following transmeridian travel (2, 5). At a physiological level, circadian misalignment can impair neuromuscular performance through several ways: depression of body temperature, dysregulation of cortisol rhythms affecting arousal and motivation, and reduced sleep quality that hampers motor memory consolidation and recovery (4, 6). These disruptions may lead to decreased central drive, impaired motor unit recruitment, and slower reaction times (6, 7). This circadian misalignment may result in fatigue, sleep disturbances, appetite loss, and gastrointestinal discomfort (5). These symptoms may lead to impairment in decision-making and reduced physical performance (3).

The adverse effects of jet lag following transmeridian travel may last from 2 to 3 days to 8–10 days (5). Additionally, compared to traveling westward, eastward travel may have a more prolonged adverse effect and require varying duration for adaptation (i.e., one day per hour difference during eastward travel compared to half a day per hour during westward travel) (8). These differences in the adverse effects of traveling eastward compared to westward are prevalent, with studies showing the negative effects lasting until the seventh day (9, 10). The combined impact of travel fatigue and jet lag affect not only sleep and other psychological feelings (e.g., feeling jet lagged) but also physical and physiological performance measures such as vertical jumps (i.e., countermovement jump and squat jump) and physiological functions such as blood pressure (e.g., blood pressure increased traveling westward and decreased traveling eastward) (9, 11). Additionally, declines in training quality manifested as impaired training performance and coordination, have also been observed (9).

In shot-put, optimal performance depends largely on upper-body strength, explosive power, neuromuscular coordination, and precise technical execution (12, 13). Even minor reductions in physical readiness or subjective well-being can impair performance due to the explosive and high-intensity nature of the event (14). For para-athletes, particularly those with impairments affecting trunk or lower-limb control, upper-body performance plays an even more central role (15). Thus, assessing travel-related changes in handgrip strength (HGS), throwing distance, perceived exertion, and well-being offers a practical and meaningful lens through which to understand the combined impact of travel fatigue and jet lag on performance in elite Paralympic athletes.

Although the impact of transmeridian travel on athletes has been studied, investigations specifically monitoring world-class para-athletes with longitudinal, sport-specific performance data are lacking (16). Previous research has examined travel effects in Paralympic populations, such as national-level wheelchair basketball athletes (17), but these studies did not focus on individual, world-class competitors in technical throwing events nor on daily performance outcomes like throwing distance. Physiological and psychological responses to long-haul travel and subsequent jet lag may differ in this population due to disability-specific factors such as altered autonomic nervous system regulation, thermoregulatory challenges, modified body composition, and potential disruptions in circadian rhythm entrainment (18, 19). For instance, athletes with spinal cord injuries may experience impaired thermoregulation and cardiovascular responses that could exacerbate travel-related stress (20). Therefore, understanding how long-haul travel and the ensuing jet lag affect para-athletes’ physical performance and well-being could be essential for optimizing training and competition strategies. Therefore, this case report aimed to assess the effects of eastward transmeridian travel (Chile to Paris) on HGS, throwing distance, subjective well-being, and perceived exertion in a World-class female Paralympic shot-putter preparing for the Paris 2024 Games.

## 2 Methods

### 2.1 Case selection and description

This case report involved a World-class female Paralympic shot-putter (age: 46.9 years; height: 168.5 cm; body mass: 69.2 kg; sport class: F54; training experience: 8 years). She holds the current World record in her sport class and won gold at the previous Paralympic Games, maintaining top global rankings since 2019, meeting criteria for World-class designation (21). The para-athlete has a traumatic spinal cord injury at the T6 level, classified as AIS B (sensory incomplete), sustained approximately 25 years prior to the study. She retains partial sensory preservation below the level of injury but lacks volitional motor control in trunk and lower limbs, requiring external trunk stabilization for seated activities such as shot-put throwing. She has a history of autonomic dysreflexia, which is managed preventively, and experiences altered thermoregulation typical of her injury level. She takes no daily medications related to her spinal cord injury and has full upper limb strength and function.

The para-athlete was instructed to maintain her programmed training routine for the duration of the study, and to avoid strenuous physical activity outside her prescribed plan (e.g., recreational sports or unsupervised exercise sessions) to minimize confounding influences on performance measures. Training load data (exercise type, volume, intensity) were recorded daily by the coaching staff and provided to the research team. The athlete's normal training during this period followed her pre-planned mesocycle aimed at peaking for the Paralympic Games, which included technical shot-put practice, strength training, and recovery sessions (e.g., hydration strategies, massage, and gradual adjustment of meal and sleep times to the destination schedule). The para-athlete was encouraged to maintain her normal hydration levels, sleep, dietary habits, and avoid any kind of drugs for the duration of the study. The para-athlete completed a medical history questionnaire and initial screening. She has not had any diseases, and she did not smoke, drink alcohol, use drugs, use sympathetic stimulants, or take any other substance potentially altering hormonal responses.

The project was conducted following the principles of the updated version of the Helsinki Statement. The athlete signed an informed consent prior to the commencement of the study. The institutional review board of the University of Los Lagos approved the study (Approval Code: 0506-024).

### 2.2 Experimental approach to the problem

To increase the ecological validity of this prospective case report, data (e.g., training loads, competitive schedule, performance outcomes) were obtained directly from the coaching staff and the participant, although they did not receive any input from the research team (22).

### 2.3 General procedures

**Figure 1:**
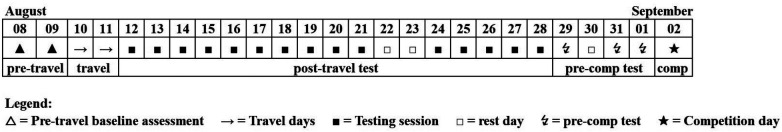
Temporal distribution of assessments relative to competition day.

The para-athlete traveled eastward across six time zones from Chile to France to attend a training camp and subsequently compete in the Paris 2024 Paralympic Games on September 2nd. The journey consisted of a commercial flight departing Santiago on August 10 around midday local time, with a 3–4 h layover at Paris Charles de Gaulle Airport (CDG), followed by two train connections to the final destination in Mulhouse. Total door-to-door travel time was approximately 24 h. Selected dependent variables were measured during 2 days before the flight, during 12 days after the flight, and during 4 days before the competition (Figure 1). The athlete was familiar with the data collection procedures. The objective performance tests (i.e., HGS, shot-put throwing distance) and the rating of perceived exertion (RPE) were assessed every day in the sports track where the para-athlete regularly trained. Subjective well-being (via the Liverpool Jet Lag Questionnaire) was self-reported by the athlete twice daily according to a prescribed schedule, as detailed below.

#### 2.3.1 Maximal isometric handgrip strength

Maximal voluntary Isometric HGS in both hands was measured using a digital dynamometer (T.K.K.5401 GRIP-D; Takei Scientific Instruments Co., Ltd., Niigata, Japan), with a measurement range from 5.0 kg to 100.0 kg, with a precision of 0.1 kg (23). Measuring procedures were performed following the recommendations of the National Health and Nutrition Examination Survey (NHANES) (24). The participant maintained the back straight against the wheelchair with her shoulders adducted ∼10°, the arm straight down the side, and the elbow extended. Measurements were taken in the morning, ∼2 h before the first training session of the day, by an experienced researcher. As the athlete was already familiar with HGS testing, no familiarization session was necessary. The athlete exerted maximum grip force for 3 s with each hand. This process was repeated 3 times with a 60-second rest between trials (25). The average of the three trials (in kilograms) for each hand was recorded as the test result*.* The test-retest reliability (intra-instrument) of TKK dynamometers has been reported as excellent, with a systematic error of ≤0.3 kg (23).

#### 2.3.2 Throwing distance

Shot-put throwing performance was evaluated using a 3 kg shot-put with the athlete seated on her throwing frame firmly fixed to the ground. Before evaluation, a warm-up of ∼5 min of dynamic mobility drills and light bodyweight exercises were conducted. The evaluation followed a simulated competition format with 3 warm-up throws followed by 6 trials. Adhering to the World Para Athletics regulations, the athlete had rest periods of 1 min between attempts (26). All six attempts were evaluated sequentially: after each throw, a marker was used to indicate the landing point, and the distance was measured using a track and field fiberglass tape. The best result achieved (meters) was recorded.

#### 2.3.3 Well-being

The Liverpool jet lag questionnaire (LJQ) (27) is a valid and reliable tool to assess jet lag-related well-being variables (3), and was used to measure the athlete's perception of sleep, alertness, and overall well-being, using visual analogue scales. Jet lag was calculated with a 0 (no jet lag)—10 (very severe jet lag) scale, while the rest of the LJQ items (sleep, fatigue, function, diet, and bowel activity) were evaluated on a −5 to +5 scale, with 0 representing ‘normal’ pre-travel levels. Higher absolute values (toward −5 or +5) indicate greater symptom severity (28).

The athlete self-administered the questionnaire twice a day: once in the morning <1 h after waking, and at night <1 h before going to bed. Items related to jet lag and fatigue were assessed both at morning and at night times, while questions about sleep were only asked in the morning, and questions about diet, function, and bowel activity were only asked just before bedtime (29).

#### 2.3.4 Perceived exertion

Internal training load was evaluated in each training session using the RPE 10-point Borg scale, ranging from 0 to 10 in 0.5-point increments, where 0 = rest and 10 = maximum effort (30). The RPE was collected after 30 min of the completion of the training sessions (31). The RPE was multiplied by training session duration (minutes) to calculate the session RPE (s-RPE), an estimation of the athlete's internal training load (ITL), expressed in arbitrary units (AU) (30, 32).

### 2.4 Statistical analysis

Descriptive statistics were used to summarize the data, including means and standard deviations (SD), and standard error of the mean (SEM) for continuous variables. To identify practically meaningful changes, the smallest worthwhile change was calculated as 0.2 multiplied by baseline standard deviation (33). Spearman's rank correlation was used to examine associations between intra-individual changes in independent variables (i.e., HGS, s-RPE) and throwing performance across the study period. Subsequently, the Rho (*ρ*) value was interpreted as follows: <0.1 (trivial), 0.1–0.3 (small), 0.3–0.5 (moderate), 0.5–0.7 (large), 0.7–0.9 (very large), and >0.9 (nearly perfect) (33). A level of *α* = 0.05 was used to determine statistical significance. Performance changes exceeding the MWC threshold were considered relevant. All statistical analyses were conducted using GraphPad Prism version 8.0.1 for macOS (GraphPad Software, San Diego, CA, USA).

## 3 Results

### 3.1 Handgrip strength

**Figure 2:**
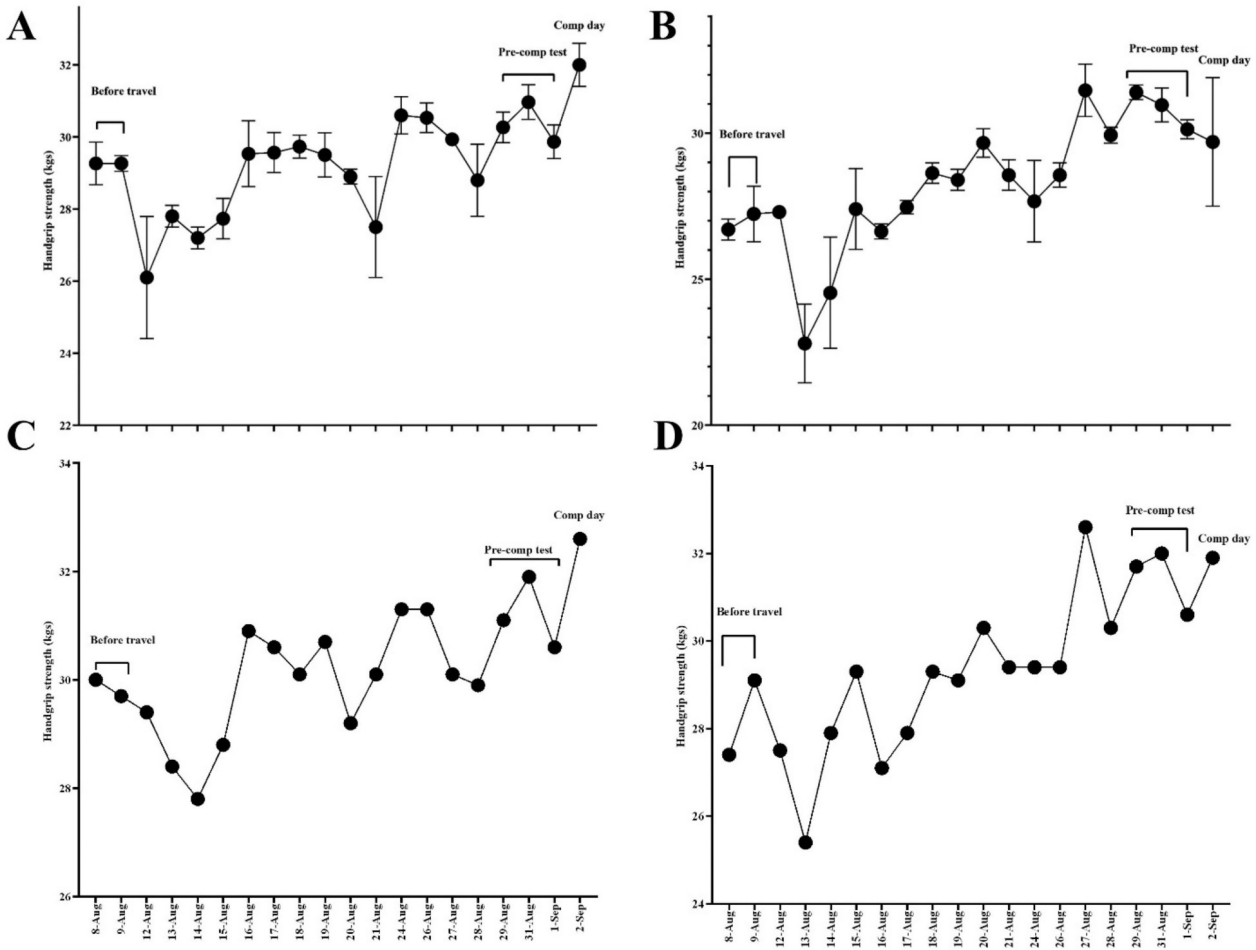
Mean and standard error of the mean for test-retest trials for **(A)** left hand and **(B)** right hand, and the highest scores obtained for **(C)** left hand and **(D)** right hand in the handgrip strength test. Note: The X-axis shows the dates on which data were collected.

The results of the HGS trials are presented in Figure 2. The daily mean ± SEM for the left and right hands are shown in panels A and B, respectively. While the maximum HGS values recorded each day are shown separately in panels C and D. Compared to the baseline (pre-travel data), left-hand HGS declined for up to four days following the eastbound transmeridian flight. In contrast, right-hand HGS remained stable on the first post-travel day but showed a decline on days two and three. Both right and left HGS values demonstrated fluctuations during the subsequent days. All observed changes in HGS exceeded the smallest worthwhile change threshold (Table 1), indicating practically meaningful fluctuations. Detailed daily comparisons of HGS relative to baseline are provided in Table 1.

**Table 1:** Longitudinal changes in handgrip strength, throwing distance, and internal training load following transmeridian travel.

Date	HGS left vs. Baseline	*Δ* Left (kg)	>SWC?	HGS Right vs. Baseline	*Δ* Right (kg)	>SWC?	Throwing *Δ* (m)	>SWC?
12-08-2024	Worse	−3.20	Yes	Better	+0.35	Yes	−0.08	Yes
13-08-2024	Worse	−1.50	Yes	Worse	−4.15	Yes	+0.08	Yes
14-08-2024	Worse	−2.10	Yes	Worse	−2.45	Yes	+0.01	Yes
15-08-2024	Worse	−1.60	Yes	Better	+0.45	Yes	+0.17	Yes
16-08-2024	Better	+0.20	Yes	Worse	−0.35	Yes	+0.42	Yes
17-08-2024	Better	+0.30	Yes	Better	+0.55	Yes	+0.15	Yes
18-08-2024	Better	+0.40	Yes	Better	+1.65	Yes	-	-
19-08-2024	Better	+0.20	Yes	Better	+1.45	Yes	+0.07	Yes
20-08-2024	Worse	−0.40	Yes	Better	+2.75	Yes	+0.22	Yes
21-08-2024	Worse	−1.80	Yes	Better	+1.65	Yes	−0.48	Yes
22-08-2024	-	-	-	-	-	-	+0.22	Yes
23-08-2024	-	-	-	-	-	-	+0.01	Yes
24-08-2024	Better	+1.30	Yes	Better	+0.75	Yes	+0.40	Yes
26-08-2024	Better	+1.20	Yes	Better	+1.65	Yes	+0.31	Yes
27-08-2024	Better	+0.60	Yes	Better	+4.55	Yes	+0.57	Yes
28-08-2024	Worse	−0.50	Yes	Better	+2.95	Yes	−0.10	Yes
29-08-2024	Better	+1.00	Yes	Better	+4.45	Yes	+0.19	Yes
30-08-2024	-	-	-	-	-	-	-	-
31-08-2024	Better	+1.70	Yes	Better	+4.05	Yes	+0.56	Yes
01-09-2024	Better	+0.60	Yes	Better	+3.15	Yes	-	-

*Δ* values: Calculated as daily mean minus baseline mean; “>SWC?”: Indicates whether the absolute change exceeded the SWC threshold (Yes = practically meaningful change); Dashes (-) indicate no data collected.

### 3.2 Correlation between HGS and performance

Spearman's correlation analysis revealed a significant positive association between left-hand HGS and throwing performance (*ρ* = 0.521, *p* = 0.032). No significant association was observed for right-hand HGS (*ρ* = 0.385, *p* = 0.127). Furthermore, session s-RPE showed a significant negative correlation with throwing performance (*ρ* = –0.567, *p* = 0.018), suggesting that higher perceived exertion during training sessions was associated with lower throwing output. The initial post-travel period (days 12-15 August) showed consistent reductions in left-hand HGS exceeding the SWC threshold (*Δ* range: −1.6 to −3.2 kg). For throwing distance, 15 of 16 measured sessions exceeded the SWC threshold (±0.01 m), with the largest decline observed on 21 August (−0.48 m) and the greatest improvement on 27 August (+0.57 m).

### 3.3 Subjective jet lag and well-being

**Figure 3:**
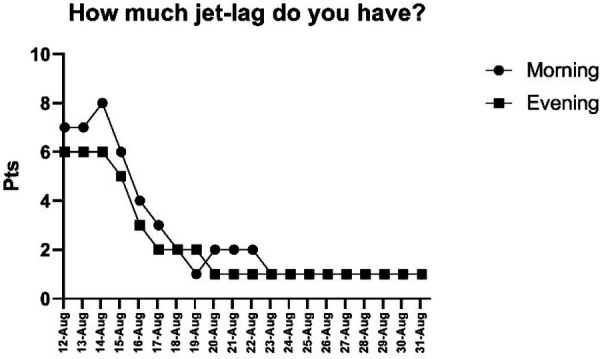
Daily assessment of jet lag symptoms as a function of time of day. Note: The X-axis shows the dates on which data were collected.

Results from the Liverpool Jet Lag Questionnaire indicated a progressive adaptation to the new time zone. Reported jet lag intensity peaked in the early post-travel days (8/10 in the morning, 6/10 in the evening), and progressively declined, stabilizing at low levels (∼1/10) by August 23 (Figure 3). Sleep-related variables showed initial variability. Difficulty falling asleep fluctuated in the first half of the monitoring period, with sustained improvements observed from day 19. Sleep onset and quality stabilized after August 20, while wake-up time variability diminished after day 16. Morning alertness, initially low, improved progressively across the observation period.

**Figure 4:**
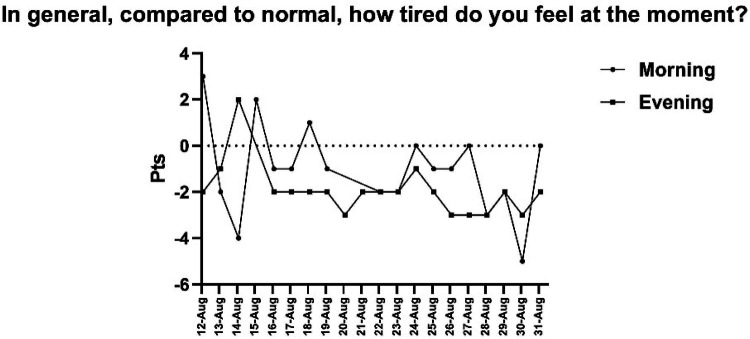
Daily score of perceived fatigue in relation to the usual level, differentiated by time of day. Note: The X-axis shows the dates on which data were collected.

Perceived fatigue was consistently higher in the morning during the early days, with both morning and evening scores gradually decreasing by August 19 (Figure 4). Mental state indicators followed a similar trend: concentration levels fluctuated early on but stabilized from August 18–21; motivation increased progressively from August 15 onward; and irritability remained moderate, never exceeding a + 3 score.

**Figure 5:**
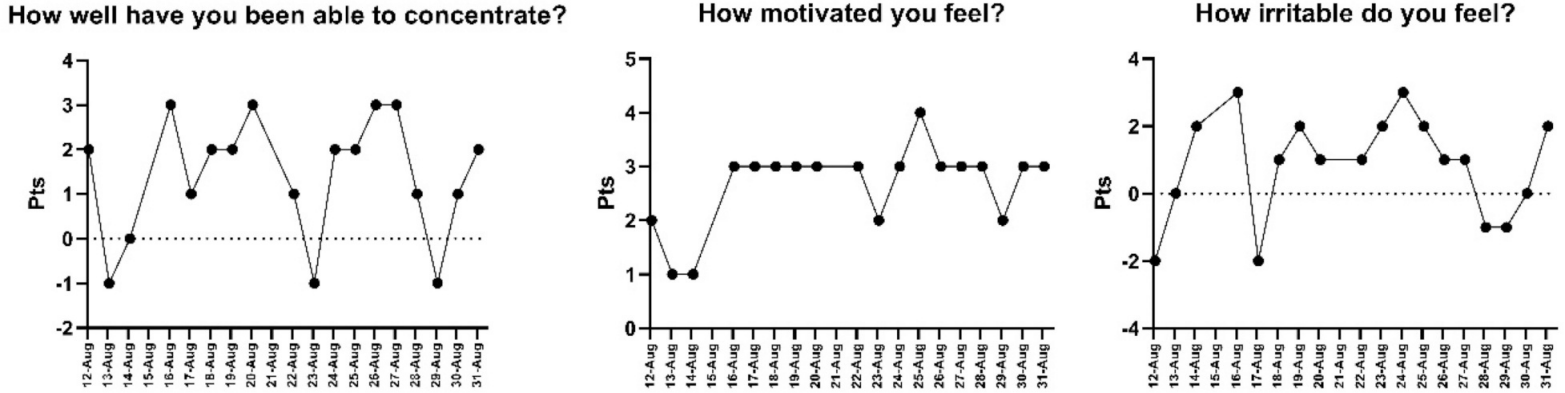
Daily evolution of mental state variables: concentration, motivation and perceived irritability. Note: The X-axis shows the dates on which data were collected.

Appetite-related responses (Figure 5) also adapted over time. Pre-meal hunger was unstable in the initial days, but stabilized between +2 and +4 by August 17. Food palatability improved during the second week, reaching up to +3. Post-meal satisfaction increased in the latter half of the monitoring period.

### 3.4 Digestive function

**Figure 6:**
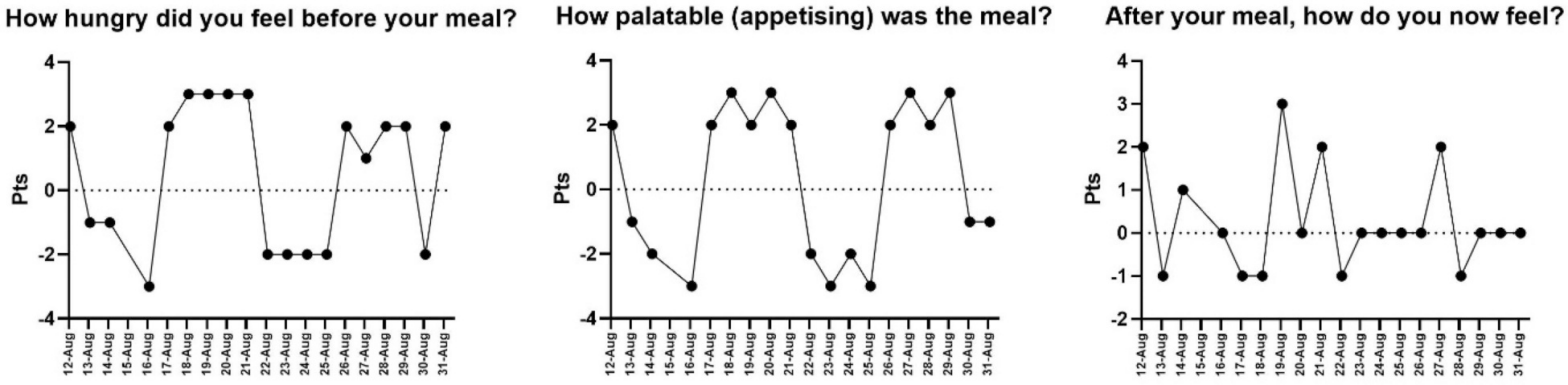
Daily fluctuations in perceived hunger, palatability, and postprandial response. Note: The X-axis shows the dates on which data were collected.

**Figure 7:**
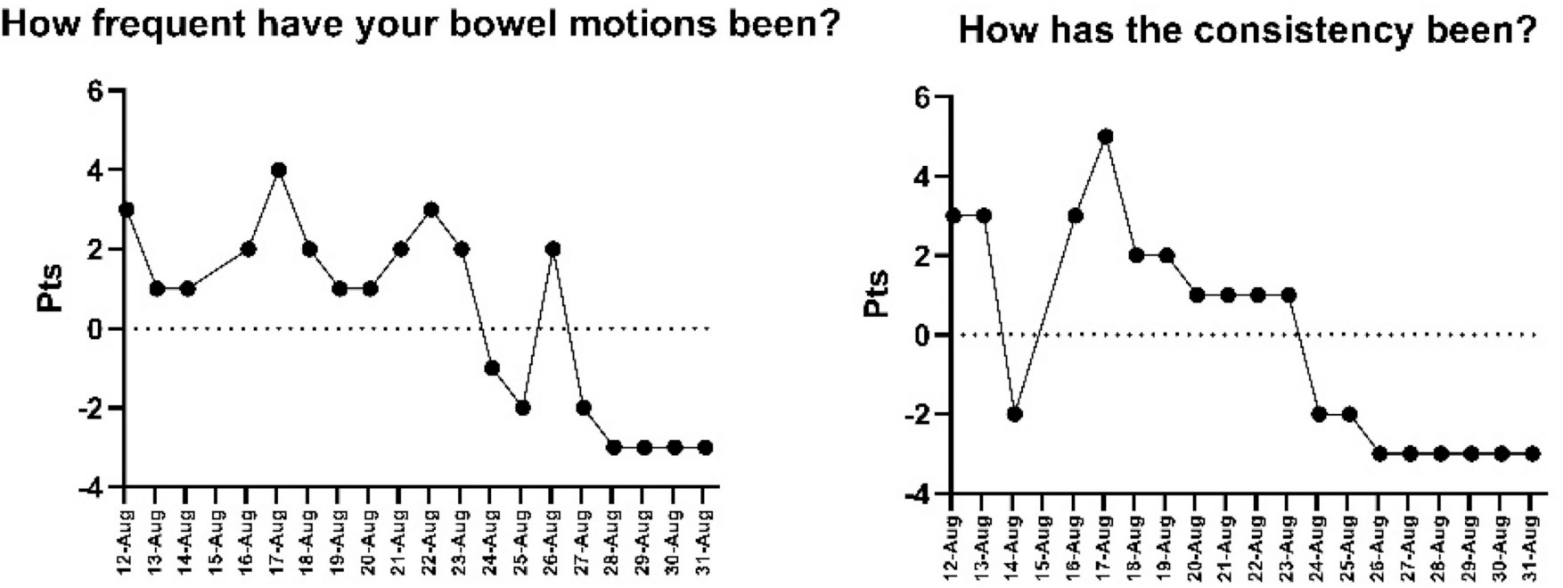
Daily variations in the frequency and consistency of bowel movements. Note: The X-axis shows the dates on which data were collected.

Digestive variables exhibited significant changes (Figures 6, 7). Stool frequency was elevated (up to +5) in the early days but dropped below baseline after day 25. Stool consistency fluctuated substantially during the first half (–3 to +5), then stabilized around −2 to −3 toward the end of the study period.

## 4 Discussion

The aim of this study was to examine the effects of long-distance eastward transmeridian travel across 6 time zones (Chile to France) on physical performance and subjective perceptions in a world-class female Paralympic shot-putter preparing for the Paris 2024 Paralympic Games. This investigation has particular relevance for the Paralympic sport, where athletes may present physiological profiles such as altered thermoregulation, body composition, or autonomic control that might increase susceptibility to jet lag and travel fatigue. Results indicated transient impairments in HGS, elevated jet lag symptoms, and moderate correlations between left-hand HGS and throwing performance (*ρ* = 0.52), as well as between s-RPE and throwing performance (*ρ* = –0.56). No significant association was found for right-hand HGS (*ρ* = 0.38).

A decline in HGS was observed during the initial 3–4 days post-travel, followed by daily fluctuations. These observed reductions in HGS were not only statistically detectable but also exceeded the minimal worthwhile change threshold, confirming their practical relevance for athletic performance readiness. Recent syntheses suggest that both eastward and westward travel can impair neuromuscular performance, with effects on strength and power potentially lasting several days (34). Although specific research in para-athletes is scarce, these findings align with previous studies in able-bodied athletes, where long-distance travel was shown to impair neuromuscular function (35). Similarly (11), reported a significant reduction in jump height immediately after transmeridian travel. Together, these results support the notion that long-haul flights may acutely impair neuromuscular performance (34), an especially relevant consideration in elite competition contexts like the Olympic and Paralympic Games.

The moderate association observed between left-hand HGS and throwing performance suggests that neuromuscular readiness could be a sensitive indicator of performance status during the travel recovery period. Although asymmetry in HGS is often observed even in elite throwers (36), the dominance of the non-throwing arm in explaining performance in this case may reflect fatigue-related compensation or reduced bilateral coordination post-travel. These findings emphasize the need to consider limb-specific neuromuscular assessments in travel-sensitive periods, especially in sports like shot-put, where upper-limb strength and coordination are critical to performance. The utility of HGS as a proxy for central fatigue and general muscle function has been recognized in both athletic and clinical populations (37), and this study supports its applicability in Paralympic contexts as well. Furthermore, the observed fluctuations in HGS over the two-week period reinforce the importance of longitudinal monitoring rather than isolated testing for travel-related assessments.

In parallel, the inverse correlation between s-RPE and throwing performance reinforces the predictive value of internal load metrics in high-performance environments. Elevated s-RPE has previously been associated with fatigue accumulation, neuromuscular inefficiency, and mood disturbances (38), all of which may contribute to suboptimal performance if unmanaged. In this case, the ability of a simple perceptual scale to reflect real performance variance underscores its value for coaches working with elite para-athletes, where physiological feedback tools may be limited or impractical in the field.

Subjective well-being assessed through the Liverpool Jet Lag Questionnaire revealed early disruptions that gradually resolved. Jet lag and fatigue were elevated immediately after travel, stabilizing within the first week. Sleep quality, alertness, and mental state improved progressively, with most variables normalizing by day 19. These patterns are consistent with literature indicating that eastward travel requires greater adaptation time, potentially one day per hour of time zone difference (3, 5). Recent pilot interventions in elite athletes suggest that implementing structured, timing-based sleep schedules upon arrival can help stabilize sleep patterns and improve sleep efficiency during the critical first days after long-haul travel (16). Moreover, the progressive yet variable resolution of different symptoms (e.g., gastrointestinal disruptions outlasting perceptual fatigue) highlights that there is no universal recovery timeline. This variability underscores a key point in recent critical reviews: outcomes are modulated by factors such as travel direction, the specific metrics assessed, and the timing of testing relative to the athlete's endogenous rhythm (34).

Gastrointestinal function exhibited persistent disruption. Stool frequency and consistency varied considerably, particularly during the latter part of the observation period. These effects may be influenced by in-flight meals, altered meal timing, and hydration challenges (39, 40). For elite para-athletes, who may already face physiological complexities, digestive health warrants greater attention (41). Future research should further investigate gastrointestinal adaptation following long-haul travel in this population. The moderate inverse correlation (*ρ* = −0.56) between s-RPE and throwing performance underscores the value of monitoring internal load metrics following transmeridian long-distance travel. From a practical standpoint, this observation highlights the need to implement training adjustments during the re-acclimatization phase. Such modifications are essential to prevent excessive fatigue accumulation and potential performance decline (8, 42). In the context of athletic training, it becomes imperative for practitioners to consider adjusting key training components (such as intensity and volume) based on subjective indicators like RPE. This approach is particularly relevant in the days immediately following transmeridian travel, as it facilitates optimal recovery for the athlete (3, 43).

This study is not without limitations. First as a single-subject case report, the findings cannot be generalized to other para-athletes. Second, while ecological validity was prioritized, the absence of strict control over external factors, such as dietary intake, hydration, and sleep hygiene, may have influenced the observed outcomes. Third, tehe use of self-reported instruments (e.g., RPE, LJQ) introduces the potential for subjective bias. Fourth, the absence of physiological biomarkers (e.g., heart rate variability or hormonal responses) and chronotype assessment limits mechanistic insights into circadian adaptation. Future research should incorporate chronotype evaluation in Paralympic athletes, as individual circadian preferences may significantly influence adaptation strategies and recovery timelines following eastward transmeridian travel. Finally, travel effects are confounded with accumulated training fatigue. Furthermore, while we focused on athlete responses, future studies should also examine how transmeridian travel affects coaching and performance staff, whose recovery is equally critical for team success (42).

Despite these limitations, our study provides several strengths and novel contributions. It provides rare, high-resolution longitudinal data on a world-class Paralympic athlete during real-world preparation for a major Games. The daily monitoring of both objective (HGS, throwing distance) and subjective (RPE, jet lag symptoms) measures enabled a multidimensional assessment of travel-induced disturbances. Methodological rigor was enhanced through validated tools (NHANES HGS protocol, Liverpool Jet Lag Questionnaire). To our knowledge, this represents the first investigation of transmeridian travel effects in a Paralympic athlete using sport-specific metrics, addressing a critical gap in the literature and offering practical insights for the Paralympic sports community.

## 5 Conclusion

Transient impairments in HGS and elevated jet lag symptoms occurred, particularly in the initial 3–4 days post-travel, with gradual adaptation over time. Left-hand HGS and s-RPE demonstrated moderate correlations with throwing performance, highlighting their potential utility as monitoring tools in the context of travel-induced fatigue and performance management.

Despite inherent limitations in generalizability due to the case report design, this research provides rare, high-resolution insight into the physiological and perceptual effects of travel in a world-class para-athlete under real-world competitive conditions. Coaches and support teams working with Paralympic athletes should consider implementing individualized monitoring strategies, including simple yet reliable tools such as HGS and RPE, to guide training decisions during travel adaptation periods. Future research should extend these findings by including larger para-athlete cohorts and integrating objective physiological markers to better understand the complex interplay between travel stress, training load, and competitive readiness.

## Data Availability

The original contributions presented in the study are included in the article/Supplementary Material, further inquiries can be directed to the corresponding author.
